# High-Throughput Isolation and Mapping of *C. elegans* Mutants Susceptible to Pathogen Infection

**DOI:** 10.1371/journal.pone.0002882

**Published:** 2008-08-06

**Authors:** Laura E. Fuhrman, Kevin V. Shianna, Alejandro Aballay

**Affiliations:** 1 Department of Molecular Genetics and Microbiology, Duke University Medical Center, Durham, North Carolina, United States of America; 2 Institute for Genome Sciences and Policy, Duke University, Durham, North Carolina, United States of America; University of Birmingham, United Kingdom

## Abstract

We present a novel strategy that uses high-throughput methods of isolating and mapping *C. elegans* mutants susceptible to pathogen infection. We show that *C. elegans* mutants that exhibit an enhanced pathogen accumulation (*epa*) phenotype can be rapidly identified and isolated using a sorting system that allows automation of the analysis, sorting, and dispensing of *C. elegans* by measuring fluorescent bacteria inside the animals. Furthermore, we validate the use of Amplifluor® as a new single nucleotide polymorphism (SNP) mapping technique in *C. elegans*. We show that a set of 9 SNPs allows the linkage of *C. elegans* mutants to a 5–8 megabase sub-chromosomal region.

## Introduction

The relatively simple innate immune system of *C. elegans* and the number of traits that facilitate genetic and genomic analysis using this organism have nurtured rapid advances into the understanding of *C. elegans* innate immunity during the last few years (reviewed in [1]–[5]). However, traditional methods of isolating and mapping *C. elegans* mutants exhibiting aberrant immune responses to pathogen infection are often labor intensive and time consuming.

The conditions that dictate the isolation of *C. elegans* mutants exhibiting **e**nhanced **p**athogen **s**usceptibility (*eps* mutants) or **e**nhanced **p**athogen **r**esistance (*epr* mutants) depend on the killing mechanism of a given pathogen. For example, *Pseudomonas aeruginosa* is a fast killing pathogen of *C. elegans* which allows the rapid identification of mutants deficient in proper immune response. Indeed, the first screen to identify *eps* mutants was performed using this pathogen [6]. Because wild-type animals infected with *P. aeruginosa* typically start to die at approximately 34 hours, mutant dead animals were isolated during a period of 16 to 30 hours. Since *C. elegans* eggs are not infected by *P. aeruginosa*, the candidate mutants were recovered by transferring individual dead animals containing their brood to plates seeded with non-pathogenic *E. coli*. Such an approach would not be possible with any of the various slow-killing pathogens of *C. elegans* [3]. For example, in the case of infections by *Salmonella enterica*, hermaphrodite nematodes initially exposed to the pathogen need to be transferred each day to fresh plates to avoid losing track of these initial nematodes in the morass of progeny. This is tedious and time consuming, greatly reducing the number of killing assays and mutant isolations that can be performed.

*S. enterica*, as well as other Gram negative and Gram positive pathogens, has been described to cause a persistent infection, leading to distension of the *C. elegans* intestinal lumen [7]. Specifically, a persistent *S. enterica* infection occurs within 72 hours of initial exposure to the pathogen and it correlates with the premature death of the nematode [7]. Taking advantage of this correlation, we designed a novel approach to avoid the rate-limiting steps of isolating, propagating and screening thousands of individual mutant strains for an *eps* phenotype. We used a sorting system specifically designed to automate the analysis, sorting, and dispensing of *C. elegans* by measuring the length of the nematode and the intensity of fluorescent markers. Animals infected with *S. enterica* expressing GFP were analyzed, sorted according to the profile of bacterial accumulation in the gut, and then dispensed into 96-well plates for subsequent studies and mapping.

Traditionally, *C. elegans* mutations are identified using a combination of multi-point or deficiency mapping by crossing the mutant of interest to marker strains. More recently, alternative gene mapping techniques such as restriction fragment length polymorphism (RFLP) analysis have been developed [8], [9]. These well established techniques, though dependable, can be time-consuming and limited by the number and location of markers and RFLP single nucleotide polymorphisms (SNPs). The original SNP mapping methods are often laborious and expensive because they require multiple steps including restriction digests and size separation. A fragment length polymorphism (FLP) map was recently established for the automated gene mapping of *C. elegans* mutants based on small insertions or deletions (InDels) [10], [11]. Although this mapping strategy greatly reduces the manual input required for techniques such as RFLP-SNP mapping, this system is limited by the number of polymorphisms that involve InDels. Even though InDels are ubiquitously dispersed across genomes, they constitute only 25% to 28% of the polymorphisms in the most commonly used Hawaiian mapping strain, CB4856 [8], [12]. Recently, a new cost-effective, flexible and accurate high-throughput SNP genotyping technique, called Amplifluor® was developed and optimized for use in mouse and human systems [13], [14]. We have optimized this technology to provide a fast and reliable method for mapping *C. elegans* mutants. Taken together, our results indicate that it is possible to use two high-throughput techniques to accelerate the isolation and mapping of *C. elegans* mutants susceptible to pathogen infection.

## Results and Discussion

### Isolation of *C. elegans* mutants based on the profile of *S. enterica* colonization of the intestine

Typically, *C. elegans* animals are propagated in the laboratory by feeding them *E. coli* strain OP50 grown on a relatively low osmolarity medium. *E. coli* is effectively disrupted by the *C. elegans* pharyngeal grinder and essentially no intact bacterial cells can be found in the intestinal lumen. In contrast, when *C. elegans* is fed bacterial pathogens, intact bacteria can be found within the intestine. Interestingly, pathogens such as *Enterococcus faecalis* [15], *Serratia marcescens* [16], *Yersinia pestis* [17], and *S. enterica* [7] establish persistent infections in the *C. elegans* intestine which cannot be displaced by transferring the animals from pathogen lawns to *E. coli* lawns. In the case of *S. enterica*, specifically, a high titer of *S. enterica* persists in the intestinal lumen, ultimately killing the nematodes [7].

**Figure 1:**
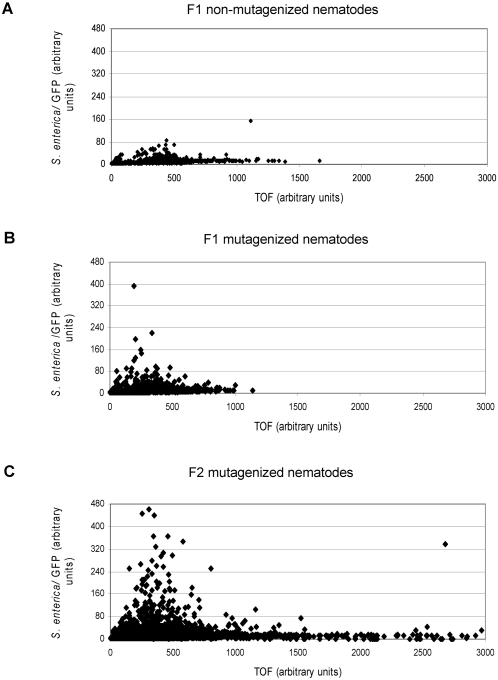
Isolation of *epa* mutants. *S. enterica*/GFP intensities of non-mutagenized and EMS-mutagenized nematodes were measured using the COPAS Biosort system. (a) First generation (F1) non-mutagenized nematodes were assayed. (b) F1 mutagenized nematodes were assayed. (c) Second generation (F2) mutagenized nematodes were assayed to isolate recessive *epa* mutants. Each group of nematodes were fed *S. enterica*/GFP for 24 hours and washed extensively prior to sorting. Both GFP intensity and TOF (time of flight; which measures nematode length) are measured in arbitrary COPAS Biosort units. One assay is shown in each case.

Taking advantage of *S. enterica* persistent infection of the *C. elegans* intestine, we have devised a strategy for identifying putative *C. elegans* mutants deficient in immune response to *S. enterica* infection by screening for mutants that exhibit **e**nhanced **p**athogen **a**ccumulation (*epa*). We used *S. enterica*/GFP to infect a mutagenized population of nematodes and used the COPAS Biosort system to isolate those mutants which exhibited high GFP intensity at a time point when wild-type nematodes exhibited low GFP intensity. In a trial experiment, F1 mutagenized and non-mutagenized wild-type nematodes were fed *S. enterica*/GFP strain SMO22 for 24 hours. After extensive washing, these two groups of nematodes were loaded into the COPAS Biosort and the **t**ime **o**f **f**light (TOF; which represents nematode length) and GFP intensity were recorded for each individual nematode. As shown in Figure 1, the non-mutagenized population showed a less dispersed pattern of bacterial accumulation, evaluated in terms of GFP intensity, than the mutagenized population. Thirteen out of a total of 23,191 first generation mutagenized nematodes displayed GFP intensities above the arbitrary baseline of 80 (0.06%), whereas only two nematodes out of a total of 10,087 (0.02%) from the non-mutagenized control group displayed GFP intensity of over 80 (Figure 1a–b). The trial experiment using F1 animals only identifies animals that have acquired a dominant *epa* mutation. Therefore, we expected to identify many more mutants in a screening using second generation (F2) mutagenized nematodes which should also help identify fixed recessive *epa* mutations. Indeed, 116 out of a total of 25,590 (0.45%) F2 mutagenized nematodes displayed GFP intensities above the arbitrary baseline of 80 (Figure 1c), whereas only one nematode out of a total of 10,087 (0.01%) from the non-mutagenized control group had a GFP intensity of over 80 (Figure 1b).

**Figure 2:**
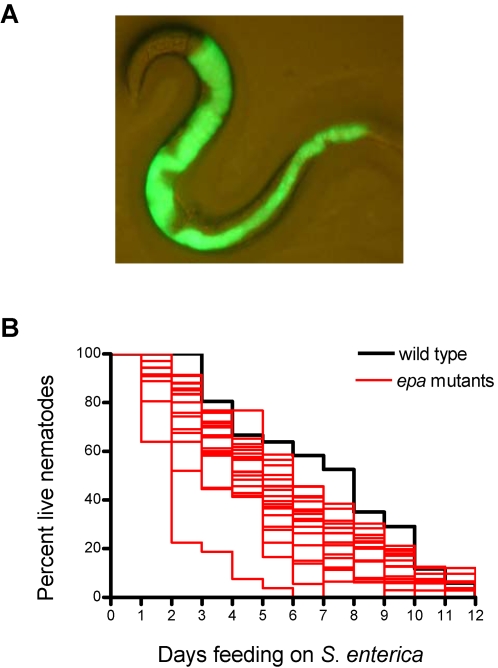
*S.enterica*/GFP colonization and pathogen susceptibility of *epa* mutants. *C. elegans epa* mutants were analyzed for *S. enterica*/GFP colonization (a). Nineteen mutants from a subset of 25 mutant strains assayed displayed an enhanced susceptibility to killing by *S. enterica* (b).

All 116 *epa* mutants exhibiting high GFP intensity were isolated to individual wells in 96-well plates and allowed to propagate for further studies. Of the 116 *epa* mutants isolated, 41 mutants successfully propagated. The bacterial accumulation of each isolated mutant was confirmed using the fluorescence stereomicroscope. While most of the *epa* mutants showed accumulation of *S. enterica* expressing GFP in the intestinal lumen (Figure 2a), in a limited number of cases, *S. enterica* appeared to be invading all the tissues of the nematode (not shown). However, additional studies demonstrated that the dissemination of *S. enterica* throughout the different tissues of the mutants was due to the internal hatching of eggs, which disrupts tissues causing matricide, rather than to specific deficiencies in responses that prevent *S. enterica* invasion such as those mediated by TOL-1 [18].

### *S. enterica* accumulation correlates with enhanced susceptibility to *S. enterica*-mediated killing

*S. enterica* persistent colonization of the *C. elegans* intestine causes a remarkable luminal distension that is similar to that observed in mammalian gastritis and that correlates with *C. elegans* death. In addition, LPS is required for *S. enterica*-mediated killing of *C. elegans*, and is also required for persistent infection of the intestinal lumen [19]. More recently, *S. enterica* pathogenicity islands-1 and -2 (SPI-1 and SPI-2), and PhoP, which are required for virulence in both mammals and nematodes, were found to be required for persistent colonization in *C. elegans* [19], [20]. These results indicate that *S. enterica* mutants in genes required for the killing of nematodes exhibit reduced colonization capabilities. This suggests that *C. elegans* mutants exhibiting enhanced accumulation of *S. enterica* may also exhibit enhanced susceptibility to *S. enterica*-mediated killing,

To address whether it is indeed possible to identify *eps* (enhanced pathogen susceptibility) mutants based on increased accumulation of *S. enterica*, a subset of 25 mutants was infected with *S. enterica* and their survival monitored throughout the complete course of the infection. Figure 2b shows 19 of 25 mutants (76%) that were killed at significantly faster rates than wild-type animals (P<0.05), indicating that the increased accumulation of *S. enterica* correlates with an enhanced susceptibility to the pathogen in these mutants.

### Validation of Amplifluor® SNP mapping for use in *C. elegans*

Traditional techniques of SNP mapping in *C. elegans* are often time-consuming and laborious. To automate the mapping of *C. elegans* mutants, we have optimized the Amplifluor® genotyping technology for SNP mapping in nematodes. This technology, which was originally developed and optimized for use in mouse and human systems [13], [14], is both flexible and accurate.

**Figure 3:**
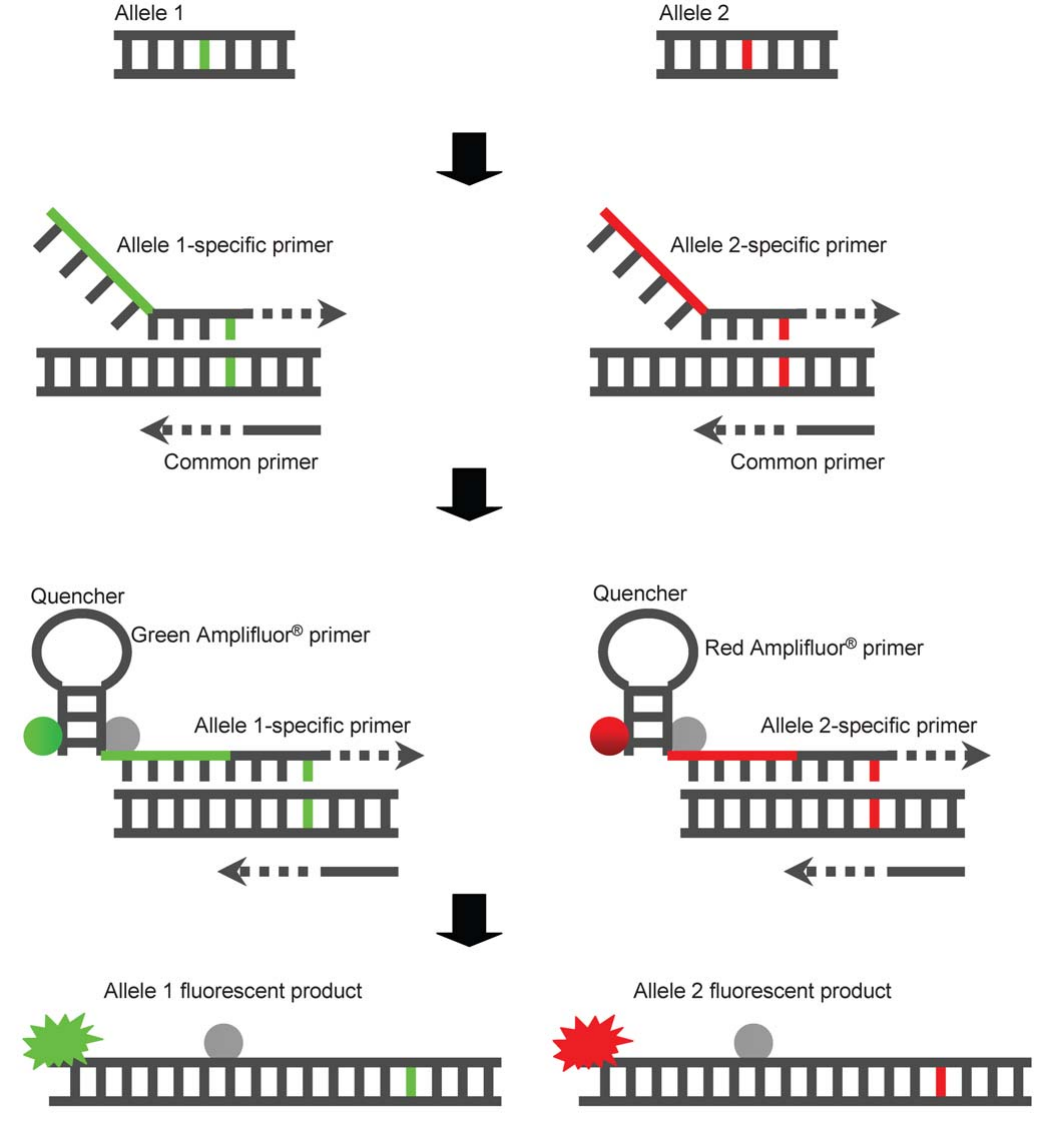
Amplifluor® technique. The two Amplifluor® primers contain a green or red fluorophore and a quencher that becomes separated when the primer is incorporated in a PCR product allowing the fluorescence to occur. The single common reverse primer and the two allele specific forward primers, which are designed to amplify across the SNP, have a 5′ tail that corresponds to one of the two Amplifluor® primers. As allele-specific PCR products are generated, the corresponding Amplifluor® primer recognizes the complement of the tail sequence and is able to prime off the newly generated PCR product. This separates the fluorophore from the quencher and generates a fluorescent signal. Homozygotes produce either a green or red signal while heterozygotes produce a yellow signal combined for both fluorophores.

**Figure 4:**
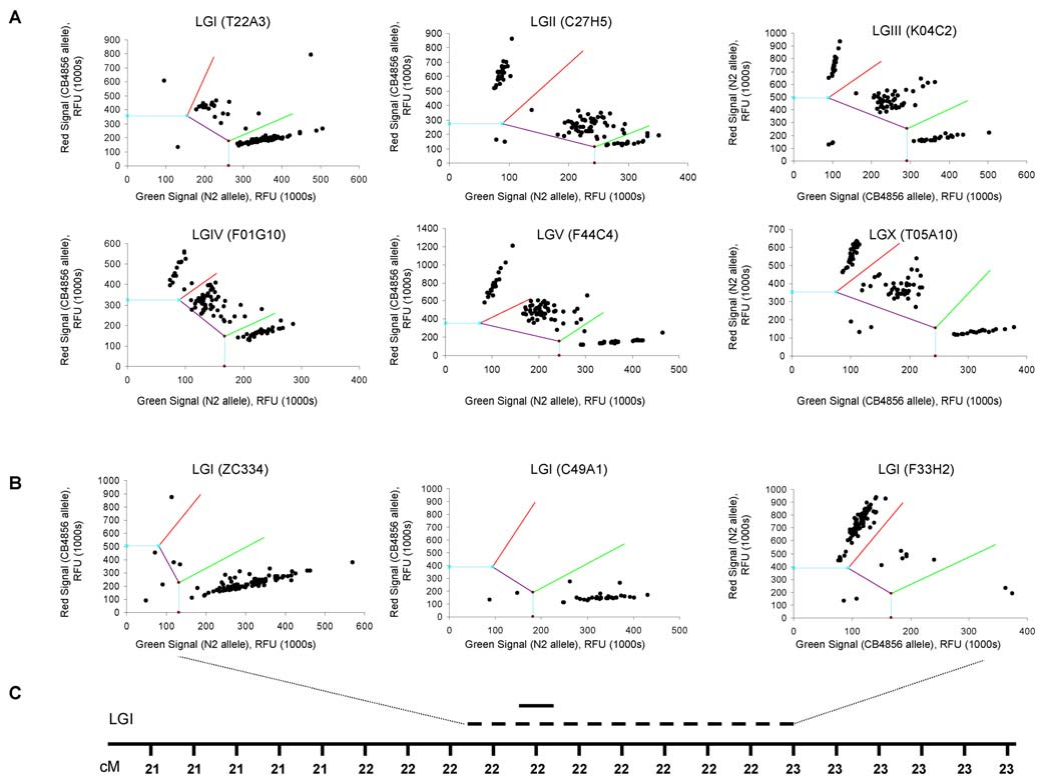
Validation of Amplifluor® SNP mapping system. Amplifluor® genotyping in *C. elegans* was validated by confirming the predicted location of the temperature sensitive mutation in *glp-4* nematodes. (a) A central SNP on each chromosome (LGI-LGX) was genotyped in 93 *glp-4* recombinant nematodes. (b) Genotype data from 3 additional SNPs on LGI accurately linked the *glp-4* mutation to the left arm of LGI between 21.70 and 23.25 cM. (c) The Amplifluor®-defined *glp-4* region is represented by the dashed line and the predicted *glp-4* region from the literature (http://www.wormbase.org) is represented by the solid line. Recombinant nematodes that are homozygous for wild type or CB4856 cluster along the red or green axes. Heterozygote nematodes cluster in the middle.

In the Amplifluor® technique, each PCR reaction mixture contains five primers: two fluorescently labeled Amplifluor® primers, two allele specific forward primers, and a single common reverse primer (Figure 3). The two Amplifluor® primers contain a green or red fluorophore and a quencher that becomes separated when the primer is incorporated in a PCR product allowing the fluorescence to occur. The single common reverse primer and the two allele specific forward primers, which are designed to amplify across the SNP, have a 5′ tail that corresponds to one of the two labeled Amplifluor® primers. As allele-specific PCR products are generated, the corresponding Amplifluor® primer recognizes the complement of the tail sequence and is able to prime off the newly generated PCR product. This separates the fluorophore from the quencher and generates a fluorescent signal. Homozygotes produce either a green or red signal while heterozygotes produce a yellow signal combined for both fluorophores. Depending upon which fluorescent label is tagged to which allele-specific primer, recombinants homozygous for wild-type N2 or CB4856 alleles cluster along the red or green axis. Heterozygote animals are represented by a cluster in the middle of the two axes (Figure 4).

First, we tested this technique by using it to map the mutation in *glp-4* nematodes, which fail to develop a germline at 25°C. We chose this mutant because the failure of *glp-4* nematodes to produce a germline at the restrictive temperature provides a rapid and simple phenotype to assay. The *C. elegans* mutant *glp-4* was crossed to the Hawaiian mapping strain CB4856 and 93 recombinant progeny exhibiting the *glp-4* phenotype were genotyped. In the initial round of gene mapping, a single SNP near the center of each chromosomal linkage group (LG) was analyzed. For LGII, LGIII, LGIV, LGV and LGX (Figure 4), the 93 recombinants were distributed according to normal Mendelian segregation for a recessive mutation, suggesting that the *glp-4* mutation is unlinked to any of these chromosomes. However, the SNP analyzed on LGI resulted in a dramatic shift of the recombinants away from the homozygous wild-type allele, suggesting linkage to LGI (Figure 4). Three additional SNPs flanking the currently predicted location of *glp-4* (http://www.wormbase.org) on LGI were used to further genotype the 93 recombinants (Figure 4). These data linked the *glp-4* mutation to a 235 Kb region on the right arm of chromosome I containing 97 predicted genes, which overlaps with the predicted *glp-4* location described in the literature (Figure 4). Although the label color varied between the wild-type N2 and mapping strain CB4856 alleles, the called genotype of each SNP in the wild-type and mapping strain controls were always correct, therefore color bias did not appear to be a problem in the Amplifluor analysis. These data indicate that Amplifluor® provides a fast and reliable method for gene mapping mutants and that it is a powerful new method for SNP mapping in *C. elegans*.

### Mapping of *C. elegans* mutant *epa-18*

**Figure 5:**
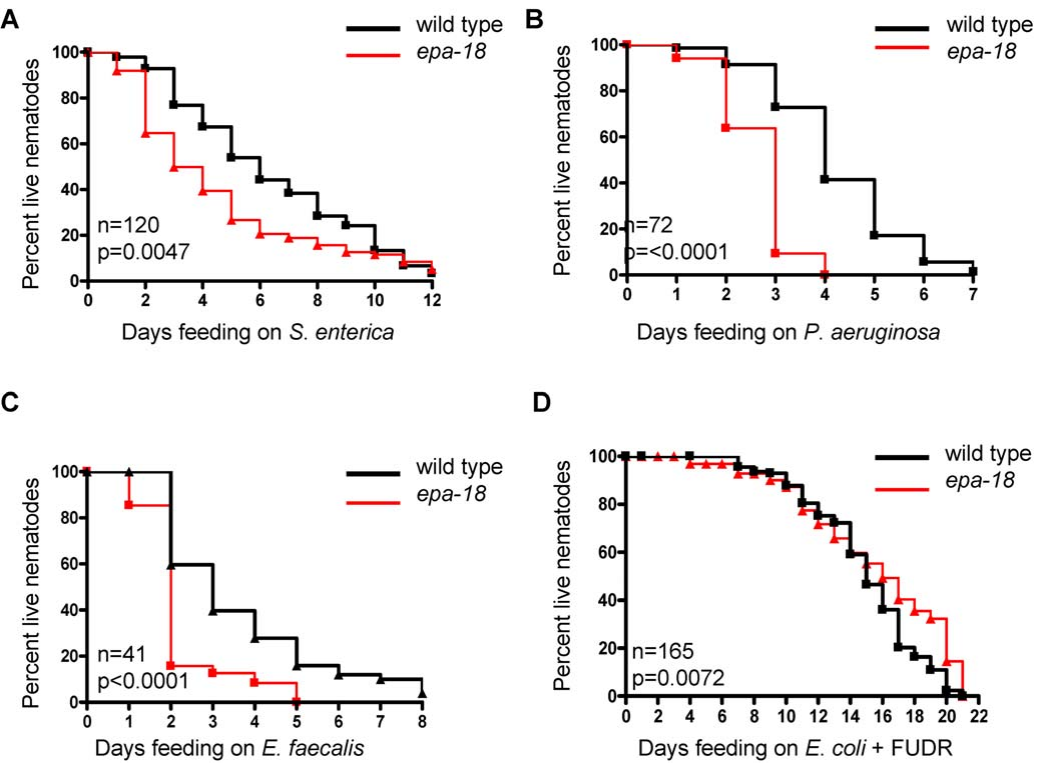
Pathogen susceptibility and life-span of *epa-18*. (a–c) Survival curves of *epa-18* and wild-type nematodes fed *S. enterica*, *P. aeruginosa*, and *E. faecalis*, respectively, confirm that *epa-18* causes an aberrant immune response to each of these three bacterial pathogens. (d) Aging assay using *E. coli* on plates containing 5′-fluoro-2′-deoxyuridine shows that the mutation does not reduce life-span.

Upon validation of the Amplifluor® SNP mapping technique for use in *C. elegans*, we chose *epa-18* mutant to begin mapping. This mutant was chosen because it appears to affect a pathway required for immunity to bacterial pathogens in general but not for survival in the absence of potentially pathogenic bacteria. As shown in Figure 5, *epa-18* animals are susceptible to infection by Gram-negative bacterial pathogens *S. enterica* and *Pseudomonas aeruginosa* and by the Gram-positive bacterial pathogen *Enterococcus faecalis*. However, the life-span of *epa-18* animals is not reduced when the animals are grown on aging plates containing *E. coli* and 5-fluoro-2′-deoxyuridine, which prevents the animals from being killed by replicating bacteria. This indicates that *epa-18* animals are specifically susceptible to live, pathogenic bacteria and that they are not sickly.

**Figure 6:**
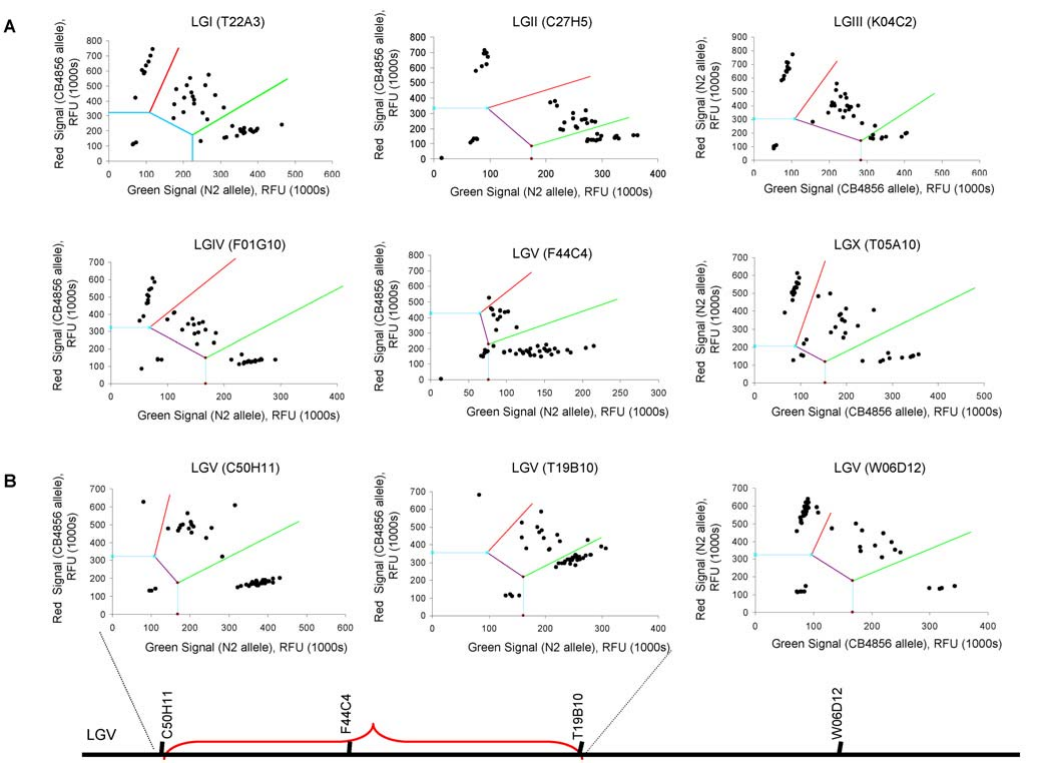
Amplifluor® genotyping data for *epa-18*. (a) A central SNP on each chromosome (LGI-LGX) was genotyped in 93 *epa-18* recombinant nematodes. (b) Genotype data from 3 additional SNPs on LGV linked the *epa-18* mutation to the left arm of LGV. Recombinant nematodes that are homozygous for wild-type N2 or CB4856 cluster along the red or green axes. Heterozygote nematodes cluster in the middle.

**Figure 7:**
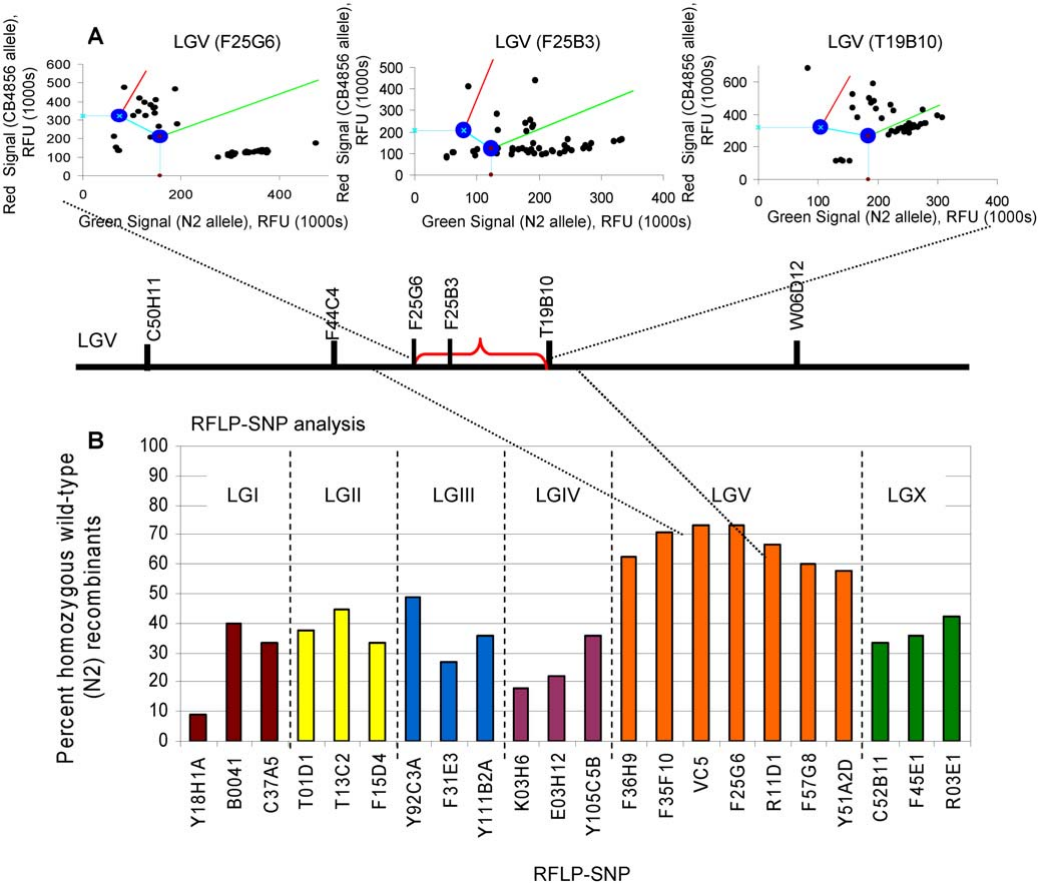
Mapping results for *epa-18* using Amplifluor® and confirmation using RFLP. (a) Four additional SNPs along LGV within the previously mapped region were used to genotype each of the 93 *epa-18* recombinant nematodes. Only data from the two most informative SNPs are shown. The relative location of each genotyped SNP is shown along the length of LGV. The region linked to *epa-18* is marked in red. (b) RFLP-SNP analysis data confirms the region defined by Amplifluor® genotyping. The percent of wild-type homozygote recombinants for each SNP is shown. The mapped region for *epa-18* using RFLP-SNP analysis overlaps with that from Amplifluor® genotyping, confirming the validity of the Amplifluor® system.

To map the *epa-18* mutation, we took advantage of the enhanced pathogen (*P. aeruginosa*) phenotype of *epa-18* mutants that is easier to track than the enhanced pathogen accumulation phenotype when the COPAS Biosort system is not used. Thus, hermaphrodite *epa-18* mutants were crossed to male CB4856 and the DNA from 93 recombinant progeny exhibiting enhanced susceptibility to *P. aeruginosa* were isolated for SNP gene mapping in a 96-well plate including 3 controls; 1) N2 DNA, 2) CB4856 DNA and 3) water template. For LGI, LGII, LGIII, LGIV, and LGX (Figure 6a), the 93 recombinants were distributed according to normal Mendelian segregation for a recessive mutation, suggesting that the *epa-18* mutation is unlinked to any of these chromosomes. However, the SNP analyzed on LGV resulted in a dramatic shift of the recombinants away from the homozygous wild-type allele, suggesting linkage to LGV (Figure 6a). In a subsequent gene mapping reaction, 3 additional SNPs on LGV were analyzed, allowing *epa-18* to be further linked to an about 8 Mb region on the left arm of chromosome V (Figure 6b). These 3 additional SNPs were chosen based on physical position on chromosome V so that four roughly equally sized segments would result. In the final round of Amplifluor® SNP mapping, 4 additional SNPs roughly equally spaced within the ∼8 Mb predicted region were analyzed. Data from the three most informative SNPs (Figure 7a) allowed mutation *epa-18* to be further linked to an about 1 Mb segment containing 222 predicted genes (Figure 7a). Thus, by taking advantage of the 93 individual recombinants, we were able to map the *epa-18* mutation to a single Mb sub-chromosomal region. Given the high number of polymorphisms present in CB4856, the limiting factor in *C. elegans* SNP mapping is the number of recombinant animals used for mapping. Genotyping a larger number of recombinants should allow for finer mapping.

In order to validate the Amplifluor® genotyping data, we used the well established RFLP-SNP analysis as an additional mapping approach. The RFLP-SNP system [8], [9], which is an accurate and inexpensive mapping methodology that does not require specialized equipment, takes advantage of polymorphisms that alter restriction enzyme recognition sites. Using RFLP-SNP analysis, a region of ∼1.5 Mb, which overlapped the Amplifluor®-mapped region, was identified (Figure 7b).

### Conclusions

Traditional methods of identifying and mapping unknown *C. elegans* mutants exhibiting aberrant immune responses to pathogen infection are often labor intensive and time consuming, greatly reducing the number of immune genes that can be identified. Here, we have devised approaches that use high-throughput methods of isolating and mapping *C. elegans* mutants susceptible to pathogen infection. We show that *C. elegans* mutants deficient in immune responses can be isolated based on an enhanced accumulation of bacterial pathogens in the intestine that can be rapidly identified using the COPAS Biosort system. In addition, we have applied the Amplifluor® genotyping to the mapping of *C. elegans* mutants and demonstrated that it is possible to link a *C. elegans* mutation to 5–8 Mb sub-chromosomal regions using only 9 SNPs.

Taking advantage of *S. enterica* persistent infection of the *C. elegans* intestine, we have devised a strategy for identifying putative *C. elegans* mutants deficient in immune response to *S. enterica* infection by screening for mutants that exhibit **e**nhanced **p**athogen **a**ccumulation (*epa*). This strategy was easily automated by using the COPAS Biosort system, allowing for the rapid screening of thousands of *epa* mutants. Further studies demonstrated that the enhanced accumulation of *S. enterica* correlates with enhanced susceptibility to *S. enterica*. In addition, the increased susceptibility to different bacterial pathogens of mutant *epa-18* indicates that the approach can be used to genetically dissect pathways involved in immune responses to pathogens in general. By coupling this approach with the Amplifluor® genotyping system for use in *C. elegans*, we provide a high-throughput method of isolation and mapping mutants deficient in immune responses. Our results also indicate that Amplifluor® genotyping is a powerful approach for the rapid mapping of any *C. elegans* mutant.

## Materials and Methods

### Mutagenesis

EMS (ethane methyl sulfonate) mutagenesis was performed as previously described [21], [22]. Briefly, the wild-type strain Bristol N2 was mutagenized with 50 mM EMS for 4 hours at 20°C as described [21], [22]. This is expected to generate ∼220 G/C→A/T transition mutations per haploid genome, →50 of which cause amino acid mutations in protein coding genes [21], [22]. From the mutagenized worms, progeny were harvested and allowed to self-fertilize in order to fix induced mutations. Non-mutagenized control nematodes were treated in an identical manner.

### Isolation of *epa C. elegans* mutants

Both mutagenized and non-mutagenized nematodes were grown to one day old gravid adults on *E. coli* strain OP50 [23] before transfer to *S. enterica/GFP* strain SMO22 [24] for 24 hours. Nematodes were harvested in M9 buffer, washed in M9 three times for ten minutes and resuspended in 30 mL S-basal buffer for 30 minutes to remove any external bacterial cells not persistently attached to the *C. elegans* intestine. To select nematodes which contained high numbers of *S. enterica*/GFP proliferating in the gut, fluorescence was measured using the COPAS Biosort (Union Biometrica) system which reports time of flight (TOF; nematode length, measured in arbitrary COPAS Biosort units) and green fluorescence (GFP-induced fluorescence, measured in arbitrary units) for each individual animal.

### *C. elegans* killing assays

*C. elegans* wild-type Bristol N2 animals and mutants were maintained as hermaphrodites at 20°C, grown on modified nematode growth medium (NGM) agar plates, and fed with *E. coli* strain OP50 as described [23]. *S. enterica* strain SL1344 [25], *Psuedomonas aeruginosa* strain PA14 [26], and *Enterococcus faecalis* strain OG1RF [27] cultures were grown in Luria–Bertani (LB) broth at 37°C. *S. enterica* and *P. aeruginosa* bacterial lawns used for *C. elegans* killing assays were prepared by spreading 20 µl of an overnight culture of bacteria on modified NGM agar (0.35% instead of 0.25% peptone) in plates 3.5 cm in diameter. *E. faecalis* bacterial lawns were prepared by spreading 20 µl of an overnight culture on brain-heart infusion (BHI) agar in plates 3.5 cm in diameter. The *S. enterica* and *E. faecalis* killing assays were performed at 25°C, and animals were scored and transferred once a day to fresh plates. *P. aeruginosa* killing assays were performed at 20°C, and animals were scored and transferred once a day to fresh plates. Animals were considered dead when they failed to respond to touch.

For the life-span assays, bacterial lawns were prepared by spreading 50 µl of a 10× concentrated overnight *E. coli* culture on modified NGM agar (0.35% instead of 0.25% peptone) containing 100 µg/ml 5′-fluoro-2′-deoxyuridine in plates 3.5 cm in diameter. Life-span assays were performed at 25°C, and animals were scored once a day. Animals were considered dead when they failed to respond to touch.

### Statistical Analyses

Animal survival was plotted as a nonlinear regression curve with the PRISM 4.00 computer program. Survival curves are considered significantly different from the control when *P*<0.05. Prism uses the product limit or Kaplan–Meier method to calculate survival fractions and the logrank test, which is equivalent to the Mantel–Heanszel test, to compare survival curves.

### Generation of Hawaiian recombinant mutant strains

For *glp-4*×CB4856 recombinant analysis, five *glp-4* hermaphrodites were placed with 10 Hawaiian CB4856 males for mating at 20°C overnight. After 24 hours, males were removed and hermaphrodite *glp-4* nematodes were isolated to separate plates and transferred to 15°C, which is the permissive temperature for *glp-4*. Thirty F1 progeny were isolated from a single successful mating and allowed to self-fertilize. Twelve F2 progeny were collected from each F1 progeny (a total of 360) and transferred to separate plates for self-fertilization. Adult F3 nematodes were phenotyped for lack of germline when grown at 25°C.

After all 360 recombinant F2 lines had begun laying eggs, each F2 animal was removed from the plate and transferred to a second plate to continue egg laying. The first set of plates was kept at 15°C to keep the lines fertile, and the second set of plates was transferred to 25°C, where the F3 progeny could be screened for loss of germline.

For *epa-18*×CB4856 recombinant analysis, five *epa-18* hermaphrodites were placed with 10 Hawaiian CB4856 males for mating at 20°C overnight. After 24 hours, males were removed and hermaphrodite *epa-18* nematodes were isolated to separate plates. Thirty F1 progeny were isolated from a single successful mating and allowed to self-fertilize. Twelve F2 progeny were collected from each F1 progeny (a total of 360) and transferred to separate plates for self-fertilization. Adult F3 nematodes were phenotyped for an increased susceptibility to *P. aeruginosa* (as described in the Methods section for *C. elegans* killing assays) and positive recombinants were used for genotyping.

### *C. elegans* recombinant analysis using Amplifluor®

Amplifluor® assays were designed and performed as recommended by the manufacturer (Serologicals, Georgia, USA). DNA lysates were generated by suspending 50 nematodes in 100 µl lysis buffer (50 mM NaCl, 10 mM Tris-Cl pH 7.5, 2.5 mM MgCl2, 0.45% Tween 20, 0.01% gelatin, 0.2 mg/ml proteinase K) and lysed at 65°C for one hour. 1 µl of this crude lysate was used as the PCR template in the PCR reaction. Each PCR reaction mixture contained five primers: two allele-specific primers, each one containing one of two different sequences at its 5′-end, and a 3′-part corresponding to the target sequence with alternating nucleotides at the 3′-end, a common reverse primer, and two fluorescently labeled Amplifluor® primers matching the 5′-portion of the allele-specific primers. The two common primers include either a green or a red fluorophore and a quencher that forms a loop in the free primer but becomes separated when the primer is incorporated in a double-stranded PCR product. A fluorescent signal is emitted only when the fluorophore and the quencher are beyond a set distance from each other. In the course of the PCR reaction, only the primers that anneal perfectly to the target DNA amplify a DNA fragment. The physical position of each SNP used in Amplifluor® mapping is shown in (Figure S1).

The DNA from each recombinant progeny exhibiting the mutant phenotype is assigned to a specific well in a 96-well plate and remains the same for each individual reaction. This makes it possible to record the genotype of each SNP analyzed for each individual recombinant progeny. Although the mutated chromosome is identified by a significant shift in allele distribution, the final candidate region containing the mutation is determined by identifying the position of the crossover event in each recombinant progeny. In other organisms, double recombination events could interfere with this type of analysis. However, in *C. elegans*, the frequency of double recombinants is essentially zero due to the nematode's highly efficient crossover interference of 1 [28]. Therefore, the chance of double recombinants is negligible and would not compromise the interpretation of the data.

### Primer design for Amplifluor® analysis

The allele-specific PCR primers and the common reverse primers were designed from each surrounding SNP sequence using Amplifluor® AssayArchitect software (http://www.assayarchitect.com/, Chemicon International, a division of Serologicals Corporation, Norcross, GA, USA). Oligonucleotides were synthesized and HPLC purified by MWG Biotech (Ebersberg, Germany). One unique 21-nucleotide tail that is complementary to the Universal Amplifluor® primer [29] was added to the 5′ end of each allele-specific primer [13]. Fluorescent measurements and data analysis for Amplifluor® were performed as previously described [29]. The sequences of the PCR primers (two allele-specific primers, and the reverse primer per SNP) used to amplify each of the SNPs are shown in (Table S1).

### Primer design for RFLP analysis

RFLP-SNPs and surrounding sequences were obtained from the *C. elegans* SNP database (http://genome.wustl.edu/genome/celegans/celegans_snp.cgi). Primers were designed using the Primer 3 program (http://frodo.wi.mit.edu/cgi-bin/primer3/primer3_www.cgi). Oligonucleotides were synthesized and HPLC purified by MWG Biotech (Ebersberg, Germany). The sequences of the primers used to amplify the surrounding sequences for each SNP are shown in (Table S2).

### PCR conditions for RFLP analysis

The same DNA lysates that were used for the PCR step during Amplifluor® analysis were used as the DNA template during RFLP-SNP analysis. 2 µl of this crude lysate was used as the PCR template. The PCR reactions contained 100 µM of each the forward and reverse primer, 125 µM dNTPs, 0.5 U Choice-*Taq*™ DNA polymerase and 1× Choice-*Taq*™ PCR buffer (Denville Scientific Inc., Metuchen, NJ, USA), in a 25 µl total volume. All reactions were performed in 96-well PCR plates sealed with an adhesive cover as follows: initial denaturation at 95°C for 2 minutes, followed by 30 cycles of 95°C denaturation for 30 seconds, 55°C annealing for 30 seconds, 72°C extension for 30 seconds, and ending with a 7 minute final extension at 72°C.

### Restriction digest conditions

All restriction digests were performed using New England BioLabs, Inc (NEB) enzymes and the recommended corresponding NEB buffer in a total reaction volume of 20 µl. A 2× digestion master mix was made as follows: 2× NEB buffer, 2× BSA, 3 U NEB enzyme to a total volume of 1 mL. 10 µl of each PCR reaction was added to 10 µl of 2× digestion master mix in a new 96-well plate and incubated at the appropriate temperature for 5 hours. Digested PCR fragments were resolved on 2% agarose gels and visualized with ethidium bromide staining.

## Supporting Information

Figure S1 Physical position of SNPs used for Amplifluor® genotyping. The physical position of each SNP was obtained from Wormbase release WS187.
(1.12 MB TIF)

Tables S1 Primer sequences, genetic location, and physical clone position of SNPs used for Amplifluor®. All primer sequences are given 5′-3′. Interpolated genetic positions were obtained from Wormbase release WS187.
(0.02 MB XLS)

Table S2 Primer sequences, genetic location, physical clone position, and restriction enzymes used for RFLP-SNP analysis. All primers sequences are given 5′-3′. Interpolated genetic positions are from Wormbase release WS187.
(0.02 MB XLS)
