# Supplementary figures and images for: High-Throughput Isolation and Mapping of C. elegans Mutants Susceptible to Pathogen Infection
Source: PLoS One. 2008 Aug 6;3(8):e2882. doi: 10.1371/journal.pone.0002882 (PMC2478710; doi:10.1371/journal.pone.0002882)

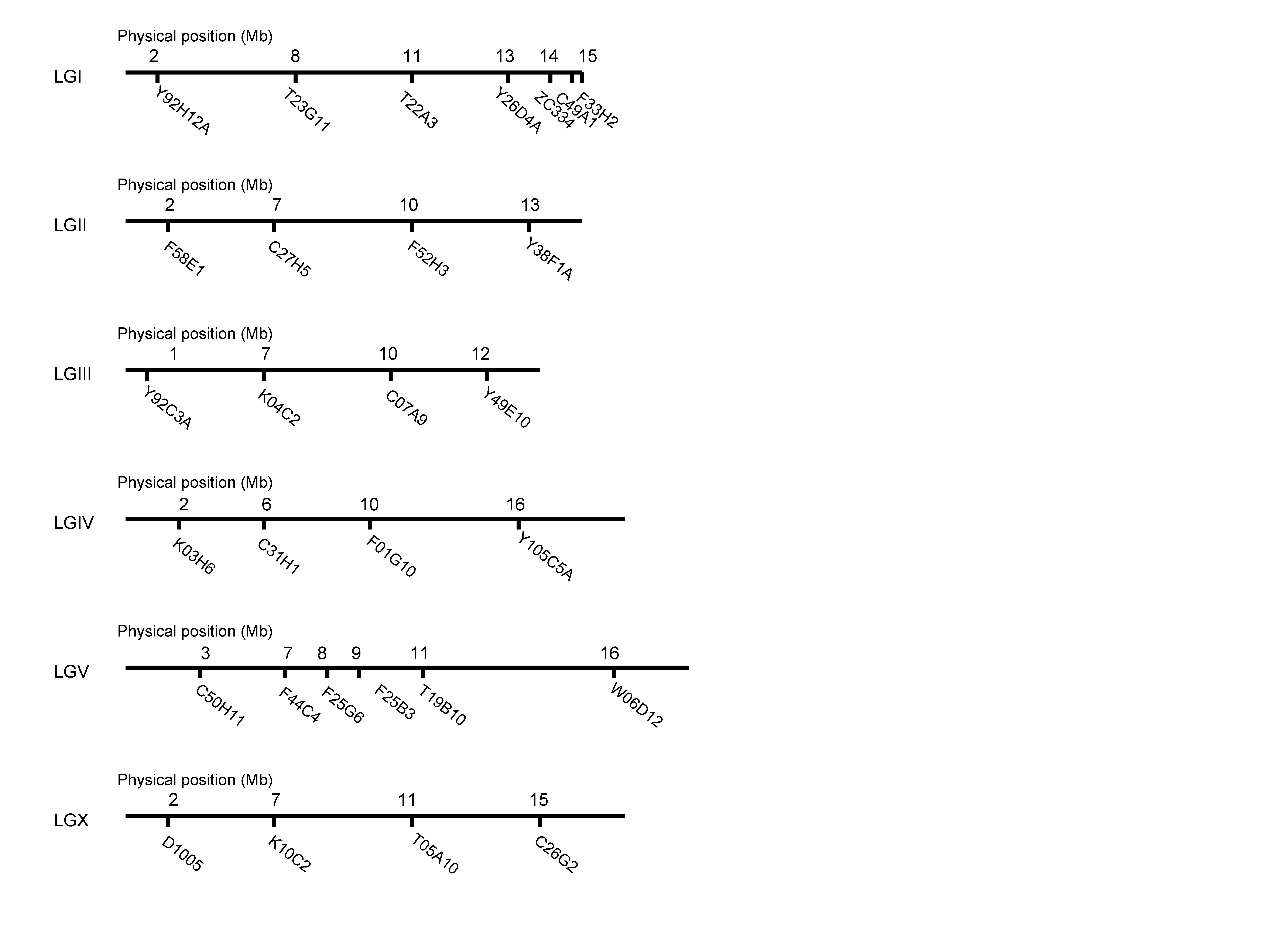

Supplement: Figure S1 — Physical position of SNPs used for Amplifluor® genotyping. The physical position of each SNP was obtained from Wormbase release WS187. (1.12 MB TIF) [file pone.0002882.s001.tif]
